# Developments in Neurotechnology and Neuroimaging: Highly Cited and Highly Viewed Articles Published in Brain Sciences in 2025

**DOI:** 10.3390/brainsci16090952

**Published:** 2026-09-07

**Authors:** Vincent P. Clark

**Affiliations:** 1 Department of Psychology, University of New Mexico, Albuquerque, NM 87131, USA; vclark@unm.edu or drvincentclark@gmail.com; 2 Energy Creating Arts, LLC, Albuquerque, NM 87122, USA

## 1. Introduction

Neurotechnology and neuroimaging are advancing rapidly and, increasingly, together. Magnetic resonance imaging (MRI) is moving from qualitative visualization toward quantitative biomarkers; electroencephalography (EEG) is being paired with machine learning to identify patterns that are difficult to recognize by conventional analysis; non-invasive stimulation is being combined with imaging to study treatment mechanisms; and smartphones and other digital technologies are extending neurological measurement beyond the laboratory. These developments are changing not only how brain function is measured, but also how neural signals may be used for diagnosis, prediction, monitoring, and intervention.

This Editorial highlights 11 papers published in 2025 in the *Brain Sciences* section on Neurotechnology and Neuroimaging that attracted substantial early attention from readers or from the scientific literature. To provide a transparent and reproducible basis for selection, papers were required to be formally assigned to the section and, when the list was assembled on 10 August 2026, to have accumulated a high number of citations and/or article views. This was used only to identify a representative group of influential contributions. Citation and view counts are dynamic, are affected by publication date and article type, and should not be interpreted as direct measures of scientific quality.

The selected contributions cover quantitative and multimodal MRI, EEG-based machine learning, brain–computer interfaces, digital biomarkers, non-invasive brain stimulation, acupuncture, and computational approaches to brain-network function. Despite their methodological diversity, several common themes emerge: the search for objective and clinically meaningful biomarkers, increasing reliance on multidimensional computational analysis, growing interest in mechanism-based intervention, and the need to establish whether sophisticated neural measurements generalize beyond the datasets and settings in which they were developed.

## 2. Quantitative and Multimodal Neuroimaging

Saltarelli et al. provide a broad review of quantitative MRI (qMRI), including relaxometry, diffusion imaging, susceptibility imaging, perfusion, myelin-sensitive methods, and volumetry. The importance of this contribution is not any single technique, but the larger transition it describes: MRI is increasingly being used to produce measurements intended to function as biomarkers rather than images interpreted primarily through relative contrast. This transition is essential for longitudinal assessment, multicenter studies, treatment monitoring, and precision medicine. At the same time, the authors emphasize that quantitative measurements become useful biomarkers only when acquisition, processing, quality control, and interpretation are sufficiently standardized across scanners, sites, and populations.

Bernetti et al. focus on the diagnostic role of MRI in subacute combined degeneration, most commonly associated with vitamin B12 deficiency. Characteristic abnormalities of the dorsal and lateral spinal cord columns can be highly informative, but overlapping patterns occur in other neurological diseases. This review is a useful reminder that an imaging abnormality rarely has diagnostic meaning in isolation. The value of advanced neuroimaging depends on its integration with clinical history, laboratory testing, and the differential diagnosis.

Liu et al. extend multimodal neuroimaging into the study of obesity, synthesizing structural, functional, diffusion, and molecular imaging evidence. Their review frames obesity as a disorder involving altered reward, cognitive control, and metabolic systems in the brain and highlights the potential for neuroimaging markers to predict or monitor interventions. This is an example of a broader movement toward precision neuroimaging, in which imaging may eventually be used not simply to describe disease-associated differences but to stratify individuals and guide treatment.

## 3. EEG, Machine Learning, and Brain–Computer Interfaces

Bai, Litscher, and Li performed a systematic review and meta-analysis of machine learning methods for EEG-based epileptic seizure detection. Across the studies included in their analysis, pooled diagnostic performance was high, demonstrating why seizure detection has become an important test case for AI-enabled neurotechnology. Equally important was the marked heterogeneity among models, preprocessing strategies, and datasets. The next step for this field is, therefore, not simply higher accuracy on established datasets but prospective validation across centers, recording systems, patient groups, and real-world clinical conditions, accompanied by improved interpretability and clinician trust.

Shi et al. developed TFSNet, a deep-learning architecture designed to integrate temporal and frequency-domain information in EEG for classification of autism spectrum disorder. The reported classification performance was impressive, and the use of cross-domain attention illustrates how modern neural networks can integrate complementary representations of electrophysiological signals. Nevertheless, the relatively small datasets highlight a recurring challenge in neurotechnology: high within-dataset performance cannot substitute for independent validation in heterogeneous clinical populations.

Zhu et al. addressed motor-imagery EEG classification for brain–computer interfaces using multidomain feature rotation and a stacking ensemble. Their results support the value of combining time, frequency and spatial information rather than relying on a single representation. Improving the robustness of motor-intention decoding remains central to practical BCIs for communication, assistive devices, rehabilitation, and ultimately closed-loop neurotechnology.

O’Reilly et al. offer a complementary perspective on EEG biomarkers. In a randomized, double-blinded, placebo-controlled study of a plant-based nootropic supplement, the investigators found changes in brain-network interdependencies without corresponding improvement in perceptual decision-making accuracy or reaction time. This dissociation is particularly instructive. As neurotechnology becomes increasingly sensitive, investigators will detect many physiological changes whose functional importance is uncertain. Demonstrating target engagement or altered network organization is valuable, but physiological change should not automatically be equated with cognitive enhancement or clinical benefit.

## 4. Neuromodulation, Acupuncture, and Mechanistic Brain Models

Sacca et al. combined transcranial direct current stimulation (tDCS), acupuncture, and arterial spin-labeling perfusion MRI in participants with chronic low back pain. The study identified distinct and partially overlapping patterns of cerebral blood flow change following real and sham interventions. This type of multimodal design is important for neuromodulation because it moves beyond the question of whether an intervention changes symptoms and asks how stimulation alters brain function. Imaging measures may eventually contribute to target selection, target engagement, dose optimization, and prediction of treatment response.

Bae et al. review the role of the hypothalamus and related circuitry in the effects of acupuncture, with a focus on pain, stress and anxiety, metabolic regulation, and addictive behavior. The paper brings together evidence involving dopamine, orexin, the hypothalamic–pituitary–adrenal axis, neuropeptide Y, and other systems. It also illustrates a broader development in stimulation and treatment research, where traditional intervention labels are increasingly being replaced by attempts to identify the neural circuits and neurochemical pathways through which treatment effects may occur.

Kang et al. approach acupuncture from a different direction, applying predictive coding and a Bayesian-brain framework to the integration of bottom-up sensory input with prior expectations and treatment context. This conceptual model is relevant well beyond acupuncture. Many neurotherapeutic interventions contain sensory, cognitive, affective, and expectancy components, and understanding their interaction may help explain individual differences in treatment response. Such models may also encourage the field to move from simple active-versus-sham comparisons toward more explicit characterization of the mechanisms contributing to both specific and contextual treatment effects.

## 5. Digital Biomarkers and Neurotechnology Beyond the Laboratory

Rudroff examines smartphone-derived digital biomarkers and artificial intelligence as tools for monitoring fatigue in neurological disorders. Fatigue is clinically important but difficult to quantify objectively, and conventional assessments provide only intermittent snapshots. Smartphone and wearable technologies can potentially capture movement, sleep, device use, cognition, voice, and ecological self-report repeatedly in daily life. The most important aspect of this approach is not the volume of data that can be collected but the possibility of validating those data against neuroimaging, physiology, standardized clinical measures, and patient outcomes.

Digital phenotyping represents an important expansion of neurotechnology. Historically, detailed assessment of nervous-system function required specialized laboratory or hospital equipment. Remote digital measures could provide longitudinal information at temporal scales that are impractical with MRI, PET, or laboratory EEG. However, useful digital biomarkers will require careful validation, attention to privacy and equity, and evidence that the measured features track clinically meaningful changes rather than merely reflecting patterns of device use.

## 6. Conclusions

The 2025 papers highlighted here illustrate a field that is becoming progressively more quantitative, computational, multimodal, and translational. MRI is evolving toward reproducible physical and biological measurements. EEG is increasingly coupled to machine learning and neurostimulation is being combined with imaging to characterize mechanisms. Also, computational models are being used to interpret both brain signals and treatment context, and digital technologies are extending neurological measurement into everyday life.

Several challenges recur across these contributions. Neuroimaging biomarkers require standardization and multicenter reproducibility. Machine learning systems require independent and prospective validation, beyond high accuracy on benchmark datasets. Neural changes detected by EEG and other forms of imaging must be related to meaningful behavior or clinical outcomes. Neuromodulation studies require better understanding of dose, targeting, individual variability, and the relationship between physiological engagement and therapeutic effect. Digital biomarkers must demonstrate validity, clinical usefulness, security, and fairness.

The most promising direction may be convergence among multimodal approaches. Quantitative imaging can define anatomy and physiology, EEG can characterize rapid changes in neural states, artificial intelligence can integrate high-dimensional information, and stimulation can be used to perturb or modify selected circuits to produce specific benefits. The long-term objective of this body of work is not simply to produce more detailed measurements of the brain, but to convert those measurements into reliable tools for prediction, diagnosis, individualized intervention, and monitoring, all ultimately leading to improved treatment efficacy with reduced side effects to reduce the suffering caused by brain and mental illness and to improve quality of life overall. The selected papers published in Brain Sciences during 2025 provide a useful view of the progress toward these goals.

## Data Availability

No new data were created or analyzed in this study. Data sharing is not applicable to this article.

